# Fluoxetine increases brain MeCP2 immuno-positive cells in a female *Mecp2* heterozygous mouse model of Rett syndrome through endogenous serotonin

**DOI:** 10.1038/s41598-021-94156-x

**Published:** 2021-07-19

**Authors:** Claudia Villani, Mirjana Carli, Anna Maria Castaldo, Giuseppina Sacchetti, Roberto William Invernizzi

**Affiliations:** Laboratory Neurochemistry and Behavior, Neuroscience Department, Istituto di Ricerche Farmacologiche Mario Negri IRCCS, Via Mario Negri 2, 20156 Milan, Italy

**Keywords:** Neuroscience, Diseases of the nervous system, Neurological disorders, Pharmacology, Pharmacodynamics

## Abstract

Motor skill deficit is a common and invalidating symptom of Rett syndrome (RTT), a rare disease almost exclusively affecting girls during the first/second year of life. Loss-of-function mutations of the *methyl-CpG-binding protein2* (*MECP2*; *Mecp2* in rodents) gene is the cause in most patients. We recently found that fluoxetine, a selective serotonin (5-HT) reuptake inhibitor and antidepressant drug, fully rescued motor coordination deficits in *Mecp2* heterozygous (*Mecp2* HET) mice acting through brain 5-HT. Here, we asked whether fluoxetine could increase MeCP2 expression in the brain of *Mecp2* HET mice, under the same schedule of treatment improving motor coordination. Fluoxetine increased the number of MeCP2 immuno-positive (MeCP2^+^) cells in the prefrontal cortex, M1 and M2 motor cortices, and in dorsal, ventral and lateral striatum. Fluoxetine had no effect in the CA3 region of the hippocampus or in any of the brain regions of WT mice. Inhibition of 5-HT synthesis abolished the fluoxetine-induced rise of MeCP2^+^ cells. These findings suggest that boosting 5-HT transmission is sufficient to enhance the expression of MeCP2 in several brain regions of *Mecp2* HET mice. Fluoxetine-induced rise of MeCP2 could potentially rescue motor coordination and other deficits of RTT.

## Introduction

The methyl-CpG-binding protein2 (MeCP2) is a nuclear factor particularly abundant in mature neurons where it regulates the expression of other genes^1^. Under- and over-expression of MeCP2 is associated with severe neurological deficits^2, 3^. Loss-of-function mutations of the X-linked *MECP2* gene (*Mecp2* in rodents) is the leading cause of Rett syndrome (RTT) in over 95% of patients^4^. In spite of research efforts aimed at identifying effective therapies, no cure is yet available. Delivery of a functional copy of *MECP2* using viral vectors, reactivation of the silenced X chromosome carrying the healthy *Mecp2* allele, and MeCP2 protein substitution have been the subject of intensive research in recent years^5–7^ and are among the most promising therapeutic approaches to this monogenic disease. In mice carrying *Mecp2* gene deletions gene replacement even in adult mice rescued core symptoms of the pathology^8–10^. In addition, recent data showed that partial restoration of the *Mecp2* gene is sufficient to improve motor function and prolong survival in a female mouse model of RTT^11^. However, the feasibility, effectiveness and safety of these therapies in RTT patients has yet to be demonstrated^12^. Thus, the identification of molecules able to cross the blood–brain-barrier, to restore normal *MECP2* expression and mechanisms downstream of *MECP2*, is still an attractive therapeutic alternative.

We propose fluoxetine (FLX), a selective serotonin (5-HT) reuptake inhibitor (SSRI), already available for the treatment of depression and other illnesses, as a candidate for repurposing in RTT. The potential utility of FLX in RTT is supported by several findings. FLX stimulates 5-HTergic neurotransmission, neurogenesis, synaptic plasticity and the expression of neurotrophic factors, which are all defective in RTT^13–19^. We recently reported that repeated dosing with FLX fully rescued motor coordination deficits in the rotarod and beam walking tests in female *Mecp2* heterozygous (HET) mice^20^. The effect of FLX was consistently reproduced under different experimental conditions and was mimicked by citalopram, another SSRI chemically unrelated to FLX. 5-HT depletion with the 5-HT synthesis inhibitor p-chlorophenylalanine (pCPA) abolished the rotarod improvement induced by FLX, and FLX was poorly effective in *Mecp2* null male mice, in which 5-HT synthesis is impaired^20^.

Previous studies have shown that repeated doses of FLX increased *Mecp2* gene and protein expression in the rat striatum and frontal cortex^21^ and enhanced MeCP2 levels, assessed by western blotting, in the hippocampus of Ts65Dn mice, a model of Down syndrome^14^. Therefore, it is not clear whether the motor effect of FLX is mediated solely by its ability to enhance 5-HT, to boost the expression of MeCP2 or both. It is not known whether FLX enhances the expression of MeCP2 in experimental models of RTT. The present study therefore aims to provide evidence that FLX can enhance brain expression of MeCP2 in *Mecp2* HET mice and that 5-HT is involved.

## Results

### Fluoxetine increases the number of MeCP2^+^ cells in various brain regions of ***Mecp2*** HET mice except the CA3

**Figure 1:**
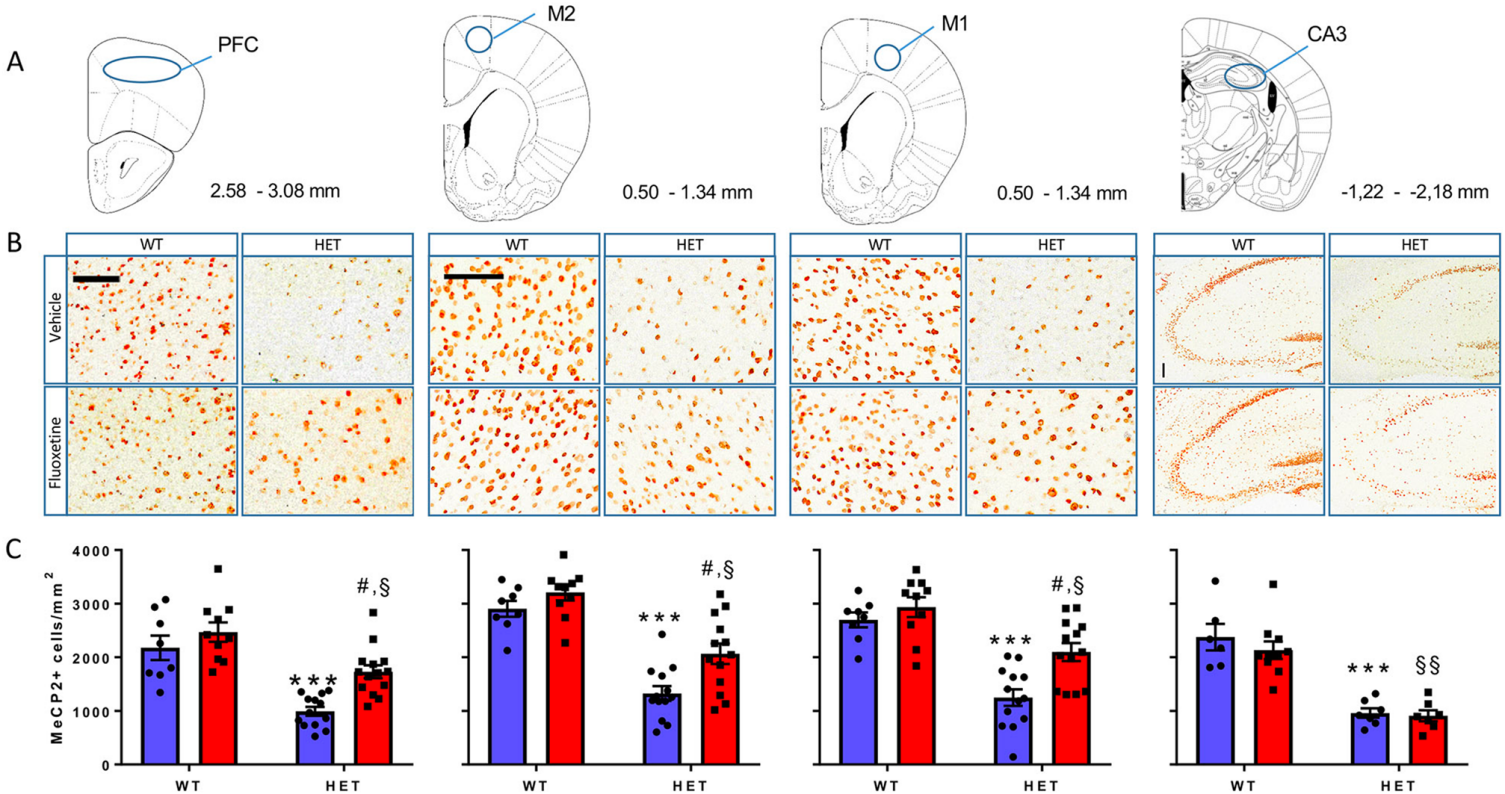
Effect of FLX on the number of MeCP2^+^ cells in the prefrontal cortex (PFC), M2, M1 and CA3. (**A**) Diagrams modified from the Franklin and Paxinos atlas^22^ (with permission) showing pre-defined areas of the mouse brain in which MeCP2^+^ cells were counted. Numbers beside the diagrams indicate the antero-posterior range of the atlas in which slices were collected. (**B**) Enlargements of the original pictures obtained by the VS120 microscope showing MeCP2^+^ cells (brown dots) in representative sections of the PFC, M1 and M2 motor cortices and CA3 of WT and *Mecp2* HET (HET) mice treated with 10 mg/kg FLX or vehicle for 14 days. On day 15 mice were euthanised and their brain processed for MeCP2 immunohistochemistry. Scale bars, 100 µm. (**C**) Histograms, generated with the Prism software (version 7.02; URL link), show the mean number (± SEM) of MeCP2^+^ cells in the PFC, M1 and M2 and CA3 of WT and HET mice (6–14 mice/group). Black dots and squares indicate individual data points. Blue and red columns indicate treatment with vehicle and FLX, respectively. ***P < 0.001 versus WT-H2O; #*P *< 0.01 versus HET-H2O; §*P *< 0.002, §§*P *< 0.0001 versus WT-FLX (Sidak’s test).

**Figure 2:**
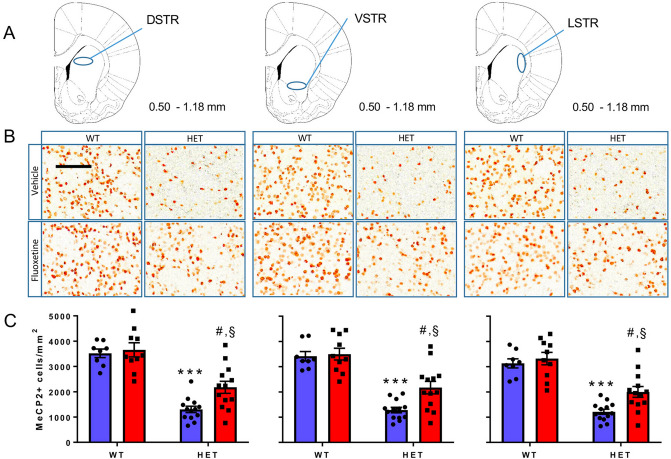
Effect of FLX on the number of MeCP2^+^ cells in the DSTR, VSTR and LSTR. (**A**) Diagrams modified from the Franklin and Paxinos atlas^22^ (with permission) showing pre-defined areas of the mouse brain in which MeCP2^+^ cells were counted. Numbers beside the diagrams indicate the antero-posterior range of the atlas in which slices were collected. (**B**) Enlargements of the original pictures obtained by the VS120 microscope showing MeCP2^+^ cells (brown dots) in representative sections of the dorsal (DSTR), ventral (VSTR) and lateral (LSTR) striatum of WT and *Mecp2* HET (HET) mice treated with 10 mg/kg FLX or vehicle for 14 days. On day 15 mice were euthanised and their brains processed for MeCP2 immunohistochemistry. Scale bar, 100 µm. (**C**) Histograms, generated with the Prism software (version 7.02; URL link), show the mean number (± SEM) of MeCP2^+^ cells in the DSTR, VSTR and LSTR of HET and WT mice (8–14 mice/group). Blue and red columns indicate treatment with vehicle and FLX, respectively. Black dots and squares indicate individual data points. ***P < 0.001 versus WT-H2O; #*P *< 0.01 versus HET-H2O; §*P *< 0.002 versus WT-FLX (Sidak’s test).

The drug’s effect on the expression of MeCP2, assessed by immunohistochemistry (IHC) in WT and *Mecp2* HET mice is shown in Figs. 1 and 2. In *Mecp2* HET mice there were significantly fewer MeCP2^+^ cells than in WT mice in all brain regions examined, reaching 46% of WT levels in the prefrontal cortex (PFC), 51% in the M1, 47% in M2 and 42% in the CA3 (Fig. 1). Two weeks of treatment with FLX partially compensated the reduced number of MeCP2^+^ cells. In the PFC, FLX restored MeCP2^+^ cells to 70% of those in WT mice treated with FLX (WT + FLX). Restoration of MeCP2^+^ cells reached 72% in the M1 and 64% in the M2. FLX had no effect in the CA3 (Fig. 1).

In striatal sub-regions of *Mecp2* HET mice, MeCP2^+^ cells were reduced to 37–38% of WT values (Fig. 2). FLX partially restored the number of MeCP2^+^ cells reaching 59–62% of those in WT mice treated with FLX. Two-way ANOVA showed significant effects of genotype and FLX but not their interaction for all brain regions examined except CA3 where FLX had no effect (Table 1). FLX did not significantly affect MeCP2 expression in any brain regions of the WT mice (Figs. 1, 2).

**Table 1:** Two-way ANOVA comparing the effect of FLX on regional MeCP2^+^ cells in *Mecp2* HET and WT mice shown in Figs. 1 and 2.

Brain region	F value (*df*)	*P* value
**PFC**		
Genotype	42.36 (1,41)	**< 0.0001**
Fluoxetine	12.13 (1,41)	**0.0012**
Interaction	2.27 (1,41)	0.14
**M1**		
Genotype	44.02 (1,40)	**< 0.0001**
Fluoxetine	8.65 (1,40)	**0.005**
Interaction	2.19 (1,40)	0.15
**M2**		
Genotype	65.03 (1,40)	**< 0.0001**
Fluoxetine	9.64 (1,40)	**0.004**
Interaction	1.66 (1,40)	0.21
**CA3**		
Genotype	64.99 (1,26)	**< 0.0001**
Fluoxetine	0.818 (1,26)	0.374
Interaction	0.350 (1,26)	0.559
**DSTR**		
Genotype	72.22 (1,40)	**< 0.0001**
Fluoxetine	5.41 (1,40)	**0.025**
Interaction	2.86 (1,40)	0.099
**VSTR**		
Genotype	69.66 (1,40)	**< 0.0001**
Fluoxetine	5.50 (1,40)	**0.024**
Interaction	3.72 (1,40)	0.059
**LSTR**		
Genotype	68.82 (1,40)	**< 0.0001**
Fluoxetine	6.24 (1,40)	**0.017**
Interaction	2.34 (1,40)	0.13

Significant effects are highlighted in bold type.

Densitometric analysis showed that FLX increased MeCP2 staining density in the PFC of *Mecp2* HET mice reaching 69% of that in WT-FLX mice. In the M1 of FLX-treated *Mecp2* HET mice MeCP2 density reached 67% of that in WT-FLX mice (Supplementary Fig. S6). FLX increases staining density and MeCP2^+^ cell number to a similar extent, in both brain regions.

### Correlation between rotarod performance and number of MeCP2^+^ cells in various brain regions

**Figure 3:**
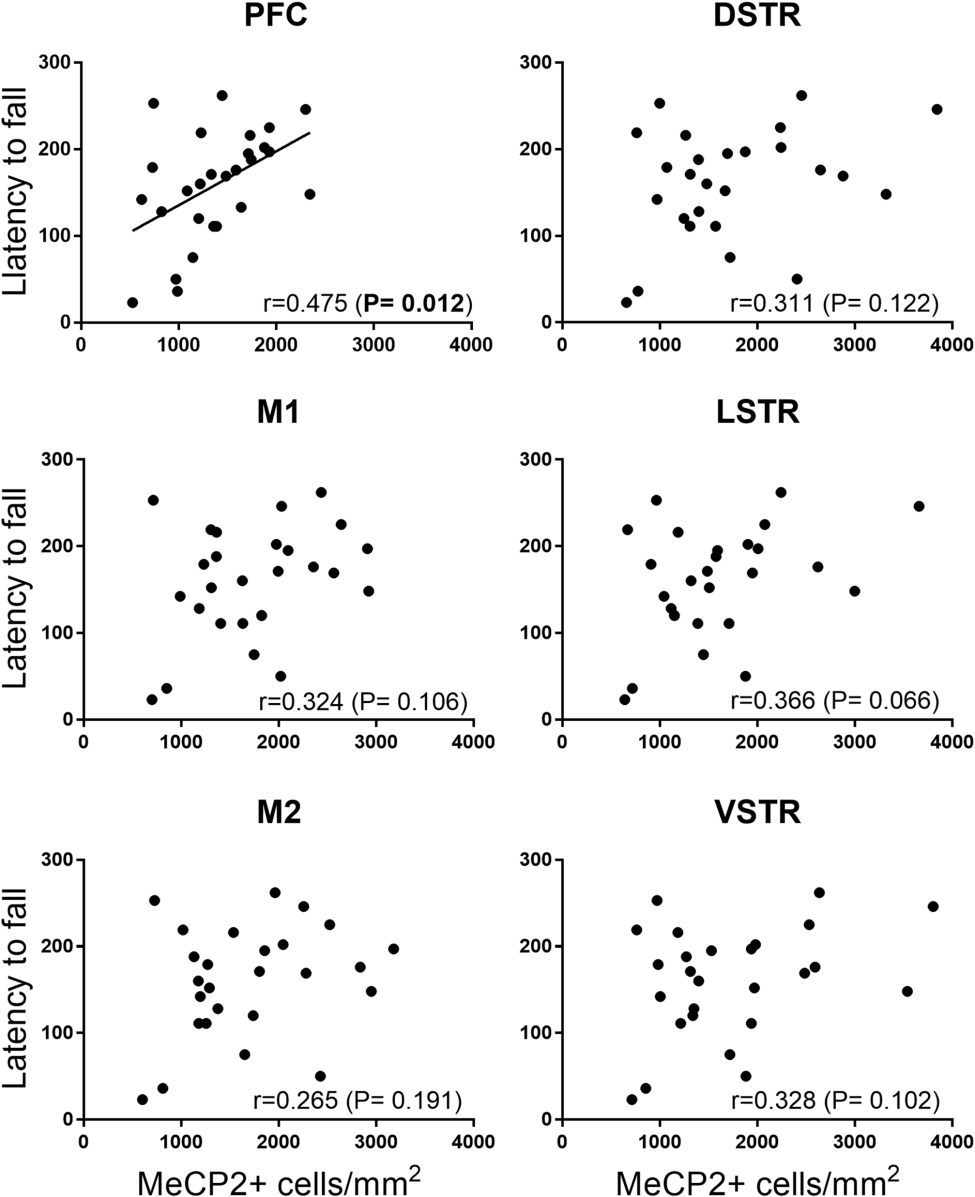
Correlation between the number of MeCP2^+^cells in various brain regions of *Mecp2* HET mice and the latency to fall from the rotarod (in seconds) assessed in the same mice. The correlation coefficient (Pearson r) associated probability and graph for each brain area were generated by the Prism software (version 7.02; https://www.graphpad.com/scientific-software/prism/). Significant correlations are highlighted in bold type. *PFC* prefrontal cortex, *M1* motor cortex 1, *M2* motor cortex 2, *DSTR* dorsal striatum, *LSTR* lateral striatum, *VSTR* ventral striatum.

Using the Pearson coefficient statistics to correlate the number of MeCP2^+^ cells and latency to fall from the rotarod assessed in the same mice^20^, a significant correlation was found in the PFC (Fig. 3). No significant correlations were found in any of the other brain regions.

### 5-HT depletion abolished the FLX-induced rise of MeCP2^+^ cells

**Figure 4:**
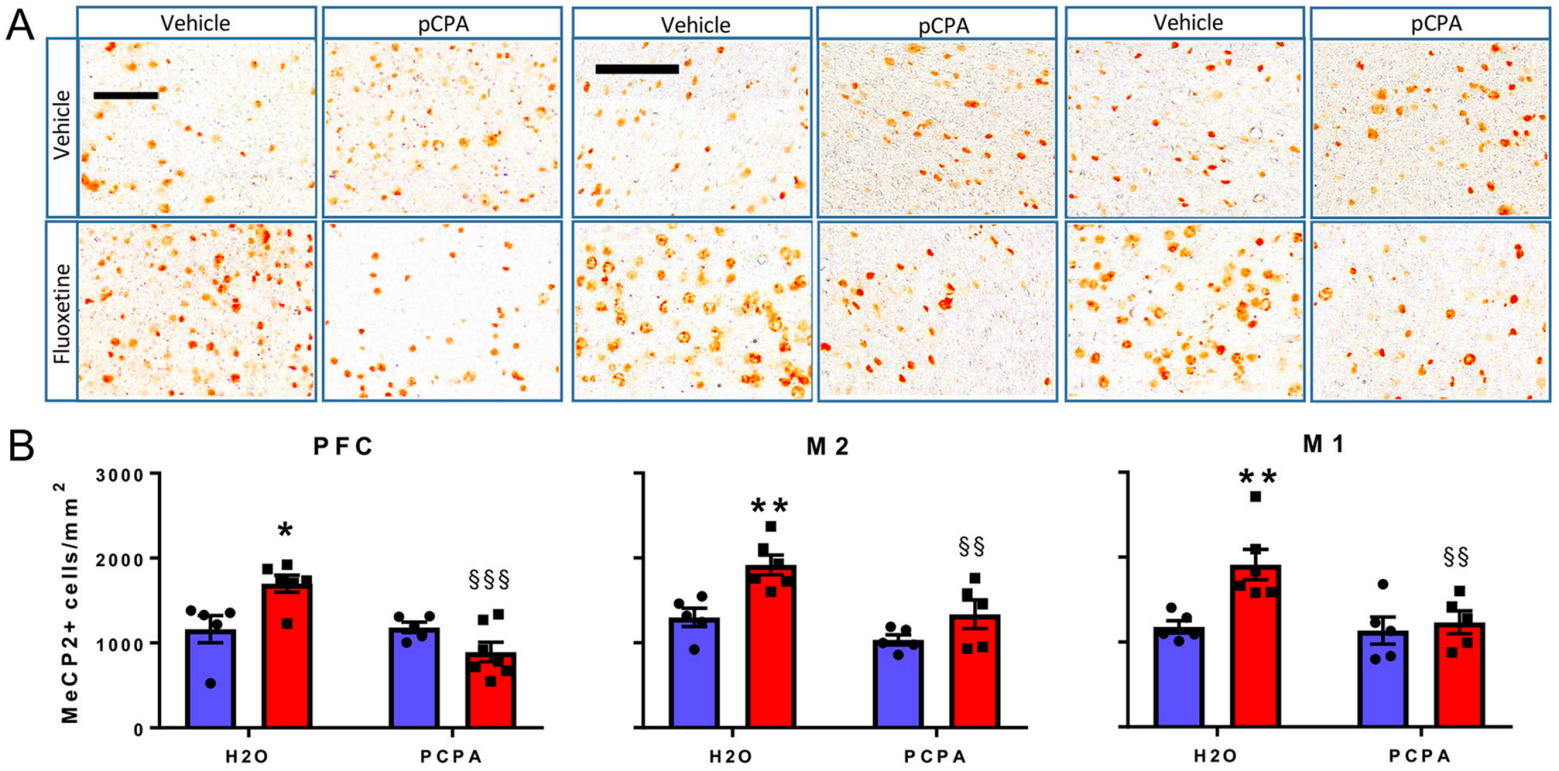
Reversal of FLX-induced rise of MeCP2^+^ cells by the 5-HT synthesis inhibitor pCPA in the PFC, M2 and M1. (**A**) Enlargements of the original pictures obtained by the VS120 microscope showing MeCP2^+^ cells (brown dots) in representative sections of the PFC, and M1 and M2 motor cortices of *Mecp2* HET mice treated with 10 mg/kg FLX or water (H_2_O) for 17 days plus PCPA or H_2_O from days 15 to 17. On day 18, mice were euthanised and their brains processed for MeCP2 immunohistochemistry. Scale bars, 100 µm. (**B**) Histograms, generated with the Prism software (version 7.02; https://www.graphpad.com/scientific-software/prism/), show the mean number (± SEM) of MeCP2^+^ cells (5–7 mice/group) . Blue and red columns indicate treatment with vehicle and FLX, respectively. Black dots and squares indicate individual data points. *P < 0.05 and **P < 0.01 versus H_2_O–H_2_O; ^§§^*P* < 0.01; ^§§§^*P* < 0.0001 versus H_2_O-FLX (Sidak’s test).

**Figure 5:**
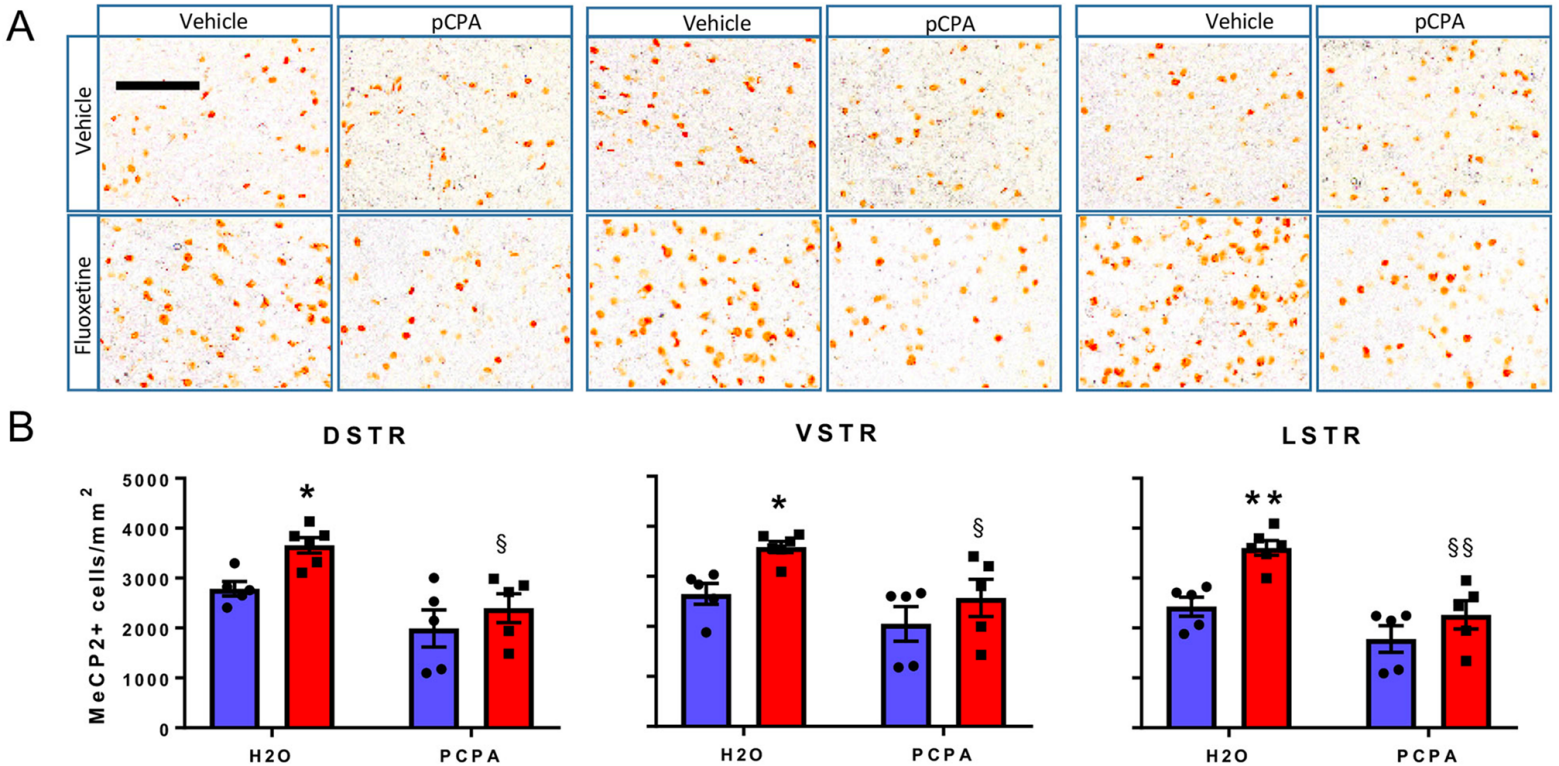
Reversal of FLX-induced rise of MeCP2^+^ cells by the 5-HT synthesis inhibitor pCPA in the DSTR, VSTR and LSTR. (**A**) Enlargements of the original pictures obtained by the VS120 microscope showing MeCP2^+^ cells (brown dots) in representative sections of the dorsal (DSTR), ventral (VSTR) and lateral (LSTR) striatum of *Mecp2* HET mice treated with 10 mg/kg FLX or water (H_2_O) for 17 days plus PCPA from days 15 to 17. On day 18, mice were euthanised and their brain processed for MeCP2 immunohistochemistry. Scale bar, 100 µm. (**B**) Histograms, generated with the Prism software (version 7.02; https://www.graphpad.com/scientific-software/prism/), show the mean number (± SEM) of MeCP2^+^ cells (5–6 mice/group). Blue and red columns indicate treatment with vehicle and FLX, respectively. Black dots and squares represent individual data points. *P < 0.05 and **P < 0.01 versus H_2_O–H_2_O; ^§^*P* < 0.05; ^§§§^*P* < 0.001 versus H_2_O-FLX (Sidak’s test).

The FLX-induced rise of MeCP2 expression was confirmed in a second cohort of *Mecp2* HET mice used to assess the role of 5-HT in the action of FLX. 5-HT depletion with pCPA abolished the increase in the number of MeCP2^+^ cells induced by FLX in the PFC, M2 and M1 (Fig. 4). The same effect was seen in the dorsal, ventral and lateral striatum (Fig. 5). Two-way ANOVA showed significant effects of pCPA and FLX (Table 2). The interaction term was significant for the PFC and M1. In mice treated with pCPA, the number of MeCP2^+^ cells did not differ between those receiving FLX or vehicle (Figs. 4, 5).

**Table 2:** Two-way ANOVA of the effect of pCPA  on FLX-induced rise of MeCP2^+^ cells in *Mecp2* HET mice shown in Figs. 4 and 5.

Brain region	F value (df)	P value
**PFC**		
pCPA	11.31 (1,19)	**0.003**
Fluoxetine	1.12 (1,19)	0.303
Interaction	12.57 (1,19)	**0.002**
**M1**		
pCPA	5.97 (1,17)	**0.026**
Fluoxetine	7.91 (1.17)	**0.012**
Interaction	4.64 (1,17)	**0.046**
**M2**		
pCPA	12.37 (1,17)	**0.003**
Fluoxetine	14.64 (1,17)	**0.001**
Interaction	1.74 (1,17)	0.204
**DSTR**		
pCPA	16.69 (1,17)	**0.0008**
Fluoxetine	6.42 (1.17)	**0.021**
Interaction	0.851 (1,17)	0.369
**LSTR**		
pCPA	19.97 (1,17)	**0.0003**
Fluoxetine	13.89 (1,17)	**0.002**
Interaction	2.416 (1,17)	0.139
**VSTR**		
pCPA	9.025 (1,17)	**0.008**
Fluoxetine	7.304 (1,17)	**0.015**
Interaction	0.612 (1,17)	0.444

Significant effects are highlighted in bold type.

### Fluoxetine did not affect MeCP2 protein levels in the whole striatum and hippocampus of WT and *Mecp2* HET mice

**Figure 6:**
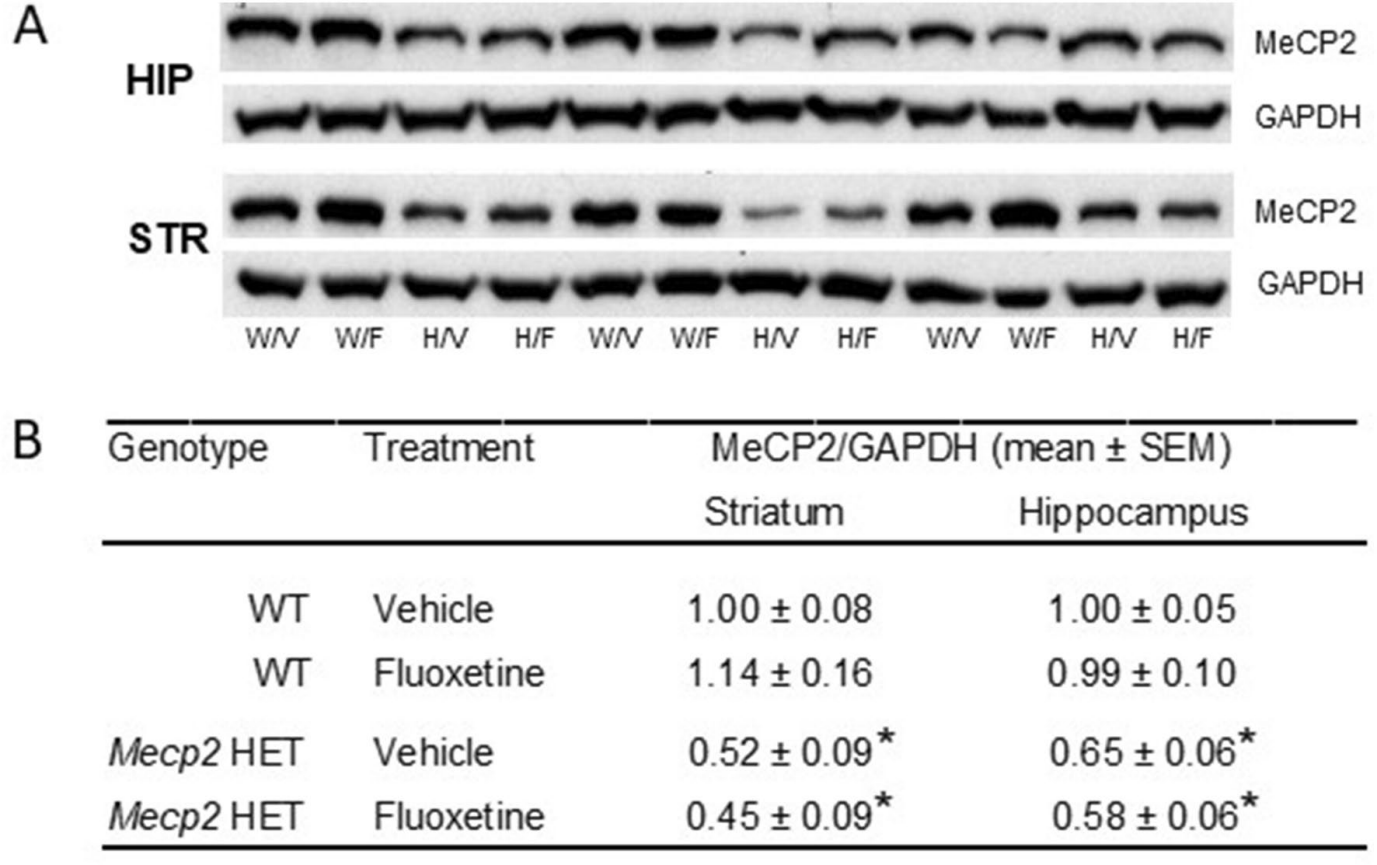
Effect of fluoxetine on MeCP2 levels in the striatum and hippocampus of WT and *Mecp2* HET mice. (**A**) Representative immunoblots (full-length blot) of the MeCP2 and GAPDH proteins. Membranes were cut approximately in correspondence to the 50 kDa MW. The half membrane containing proteins heavier than 50 kDa was incubated with primary antibody against MeCP2, while the half membrane containing proteins lighter than 50 kDa was incubated with the antibody against GAPDH. W/V and W/F indicate WT mice receiving respectively vehicle and fluoxetine. H/V and H/F indicate *Mecp2* HET mice receiving vehicle and fluoxetine. (**B**) Normalized MeCP2 expression (mean ± SEM of 5–6 mice per group) in wild type (WT) and *Mecp2* HET mice receiving fluoxetine or vehicle. *HIP* hippocampus, *STR* striatum. *P < 0.05 versus respective WT control group (Tukey–Kramer’s test).

Western blots of MeCP2 protein in tissue homogenate showed a reduction by about 50% of MeCP2 levels in the striatum and hippocampus but did not reveal any effect of FLX on protein (Fig. 6). ANOVA showed a significant effect of genotype (Striatum, F1, 20 = 28.44, *P* < 0.0001; Hippocampus, F1, 18 = 27.67, *P* < 0.0001) but not treatment (Striatum, F1, 20 = 0.11, *P* = 0.75; Hippocampus, F1, 18 = 0.29, *P* = 0.60) and genotype x treatment (Striatum, F1, 20 = 0.84, *P* = 0.37; Hippocampus, F1, 18 = 0.21, *P* = 0.65). Likewise, FLX had no effect on cortical MeCP2 levels (Supplementary Fig. S5).

## Discussion

Repeated treatment with FLX, which can rescue the motor coordination deficit in *Mecp2* HET mice^20^, increased the number of cells expressing the MeCP2 protein in the PFC, M1 and M2 motor cortices, DSTR, VSTR and LSTR, but not in the CA3 region of the hippocampus. The 5-HT synthesis inhibitor pCPA prevented the effect of FLX in all brain regions examined, demonstrating that the FLX-induced increase of MeCP2^+^ cell numbers depends on endogenous 5-HT. In line with this, 5-HT enhanced the expression of *Mecp2* in cultured PC12 cells^23^, which are capable of taking up 5-HT from extracellular medium and express 5-HT receptors on the cell membrane^24,25^.

MeCP2 protein is present in most brain neurons and regional differences in its abundance have been related to specific behavioral impairments^26–28^. We found a significant correlation between rotarod performance and the number of MeCP2^+^ cells in PFC but not in the other brain regions, suggesting that increased MeCP2 in the PFC may underlie FLX’s ability to improve rotarod performance in *Mecp2* HET mice. In line with this proposal, the role of the PFC in motor coordination is supported by a previous study in C57BL/6 mice showing that the volume of frontal association cortex, an indicator of structural plasticity, was positively correlated with better rotarod performance^29^.

Although we found no significant correlation between the effect of FLX on rotarod performance and the increase of MeCP2^+^ cells in the other brain regions analyzed, we cannot exclude the contribution of, at least, the striatum and motor cortex. This stems from the observation that the selective deletion of *Mecp2* from the striatum replicates the rotarod impairment caused by global *Mecp2* deletion, while the selective preservation of striatal *Mecp2* expression in otherwise *Mecp2* null mice was sufficient to prevent the impairment of rotarod performance^30,31^. In addition, motor cortex is critical for the acquisition, consolidation and retention of motor skill, including rotarod skill^32^.

Previous studies in conditional knockout mice have identified cell types in which the deletion or restoration of the *Mecp2* gene affected rotarod performance. Selective expression of *Mecp2* in GABAergic or glutamatergic neurons of *Mecp2*-null mice was sufficient to rescue the rotarod deficit^33,34^. Restoration of *Mecp2* expression in GABAergic neurons rescued the rotarod deficit preferentially in male mutants^34^, while the expression of the gene in glutamatergic neurons was sufficient to rescue the rotarod deficit in female *Mecp2* HET but had no effect in *Mecp2* null mice^33^. As we found that FLX was more effective in restoring rotarod performance in *Mecp2* HET than in *Mecp2*-null mice, conceivably the rise of MeCP2 in glutamatergic neurons underlies the drug’s ability to rescue this impairment.

The proposal that FLX may act in a subset of cells in which increased levels of MeCP2 is sufficient to rescue the motor coordination deficit is in line with the lack of FLX’s effect on MeCP2 protein in whole homogenates of the striatum, hippocampus and cortex—investigated by western blotting—and the expression of the *Mecp2* gene in the striatal homogenate as assessed by qRT-PCR (Supplementary Table S1). Thus, further investigation is now warranted to examine the requirement for MeCP2 in different brain regions and cell populations in FLX’s effect on motor coordination in *Mecp2* mutant mice.

FLX increased *Mecp2* gene and protein expression in the striatum and PFC of Wistar rats^21^, whereas it had no effect on MeCP2^+^ cells in any brain region of WT mice. As the FLX dose and duration of treatment were similar in our and the earlier study in the rat, it is likely that species differences influence the ability of FLX to affect MeCP2 expression.

We found that FLX had no effect on the number of MeCP2^+^ cells in the CA3 region of the hippocampus of *Mecp2* HET mice where the expression of the 5-HT transporter (*SERT*) is selectively reduced^35,36^. Reduced production of the SERT protein impairs the response to SSRIs^37^ and may account for the failure of FLX to enhance MeCP2 expression in the CA3.

The mechanism by which FLX raises the number of MeCP2^+^ cells in *Mecp2* HET mice was not addressed in the present study. FLX can only increase MeCP*2* in cells that already express the wild type allele of *Mecp2* or might reactivate the *Mecp2* gene in cells in which the wild type allele is silenced because of the X chromosome inactivation (XCI), a physiological phenomenon by which one of the two X chromosomes in female cells becomes transcriptionally silent to compensate differences in gene dosage between sexes^38^. The first mechanism is unlikely as FLX had no effect on MeCP2^+^ cells in WT mice (present study). The potential effect of FLX on XCI is supported by indirect evidences showing that FLX enhances the levels of the transforming growth factor-β1 (TGF-β1) in cultured astrocytes^39^. TGF-β1 suppresses the expression of the X-inactivating specific transcript (Xist), a long non-coding RNA which plays a key role in XCI^6,38^. Thus, FLX by increasing TGF-β1 may suppress Xist and in turn enhance the expression of *Mecp2*.

Previous reports that the stimulation of 5-HT neurotransmission with SSRIs and 5-HT receptor agonists ameliorates symptoms in *Mecp2* mutant mice^26–30^ confirm the importance of the 5-HT system as a potential therapeutic target for RTT^40^. The SSRI citalopram improves respiratory sensitivity to CO_2_ in *Mecp2*^*tm1*.1Bird^ mice^41^. The 5-HT_1A_ receptor agonists 8-OH-DPAT, F15599 and sarizotan—which is also a dopamine D2 receptor partial agonist, reduce apnea and improve breathing regularity^42–44^. However, sarizotan failed to reduce apnea in patients with RTT and did not achieve the trial’s secondary end-point, which included tests of motor function (press release); this raises questions about the validity of 5-HT_1A_ receptor stimulation as a therapeutic target for RTT (see Abdala et al.^45^ for a review of the role of 5-HT_1A_ receptors in RTT). LP-211, a 5-HT_7_ receptor agonist, improves cognitive functions and reduces anxiety in *Mecp2*-308 mice^46^. Thus, enhanced 5-HT transmission resulting from blockade of the 5-HT transporter or selective stimulation of 5-HT receptor subtypes is a common feature of serotonergic drugs that improve RTT deficits in mouse models. Mirtazapine, a noradrenergic and serotonergic antidepressant, rescues cortical atrophy in *Mecp2* null mice^47^. Although mirtazapine blocks 5-HT_2/3_ receptors, the fact that it blocks α_2_-adrenoceptors results in an increased firing rate of 5-HT neurons of the raphe, 5-HT release and tonic activation of hippocampal 5-HT_1A_ receptors^48^.

Currently, information on the effects of FLX and other SSRIs in patients with RTT is limited to a few case reports showing that these drugs may be effective on psychiatric, behavioral and motor symptoms and sleep apneas^49–51^. An open study on the effects of FLX in RTT was terminated prematurely (EudraCT Number: 2008-000787-16) and to our knowledge no other clinical assessments of the effects of SSRIs in RTT have been published. Thus, further clinical studies are needed to draw any firm conclusion about the efficacy of these drugs in patients with RTT.

As the FLX-induced improvement of motor coordination and increased number of MeCP2^+^ cells depend on endogenous 5-HT, the present data suggest that boosting 5-HT neurotransmission may be sufficient to drive both effects. 5-HT levels and *TPH2* expression are reduced in the brain of patients with RTT^52^. Thus, FLX may possibly have limited effect on motor coordination in patients. However, stimulation of 5-HT synthesis restored the antidepressant-like effect of SSRIs in mice that did not respond to the SSRI alone because of genetic polymorphism of *Tph2* reducing the biosynthesis of 5-HT or following treatment with pCPA^53–55^. These findings suggest that boosting 5-HT synthesis may improve the efficacy of SSRIs on motor coordination and increase MeCP2 expression also in subjects with impaired 5-HT synthesis.

The identification of the molecular and cellular mechanisms by which FLX boosts the expression of brain MeCP2 has the potential to provide novel therapeutic targets to counteract RTT deficits.

## Materials and methods

### Experimental design and statistical analysis

Breeding pairs consisting of female *Mecp2* HET (B6.129P2(C)-*Mecp2*^*tm1.1Bird*^/J; stock: 003890) and male wild type (C57BL/6 J; stock: 000664) mice were purchased from The Jackson Laboratory (Bar Harbor, ME). Details of mice breeding and husbandry are described in Villani et al.^20^. The first test cohort comprised 34 *Mecp2* HET and 26 WT littermate controls. They were used to assess the ability of FLX to enhance the expression of MeCP2 protein in the brain of *Mecp2* HET mice (primary end point of the study). Mice of this cohort were the same as those used to study the motor effects of FLX by Villani et al.^20^ and they were 9 weeks old at the start of FLX treatment. At the end of the behavioral assessment they were euthanised, their brain were removed and processed according to the immunohistochemical protocol described below. A second cohort of 21 *Mecp2* HET mice, 9 weeks old at the start of treatment, was used to assess the effect of pCPA on FLX-induced rise of MeCP2 expression.

All statistical analyses were performed by GraphPad Prism version 7.02 for Windows (GraphPad Software, CA) and all data were expressed as mean ± SEM of the number of MeCP2 immunopositive (MeCP2^+^) cells. Two-way ANOVA, followed by Sidak’s test was used to assess the effect of FLX on MeCP2 protein expression in WT and *Mecp2* HET mice and the effect of pCPA on FLX-induced rise in the number of MeCP2^+^ cells in *Mecp2* HET mice.

Gaussian distribution was formally tested for the effect of FLX on MeCP2 expression, the primary end-point of the study, with the D’Agostino-Pearson normality test included in the GraphPad Prism 7.02 package^56^. This analysis confirmed the normal distribution of the MeCP2^+^ cell in all experimental groups of the PFC, M1, M2, DSTR, VSTR, LSTR. Because of the small number of subjects, no normality test was applied to CA3 data. The correlation between the number of MeCP2^+^ cells and the latency to fall from the rotarod, measured in the same mice^20^ included *Mecp2* HET mice receiving vehicle and FLX, was analysed with the Pearson r correlation coefficient (Graph Pad Prism 7.02 package).

Data reported here are part of a larger project^20^ planned and conducted according to the Arrive guidelines.

### Ethical statement

The *“*Istituto di Ricerche Farmacologiche Mario Negri IRCCS” adheres to the principles set out in the following law, regulations, and policies governing the care and use of laboratory animals: Italian Governing Law (D.lgs.26/2014; Authorization n. 19/2008-A issued on March 6, 2008 by Ministry of Health); “Mario Negri” Institutional Regulations and Policies providing internal authorization for persons conducting animal experiments (Quality Management System Certificate—UNI EN ISO 9001:2008—Reg. N° 6121); the NIH Guide for the Care and Use of Laboratory Animals (2011 edition) and EU directives and guidelines (EEC Council Directive 2010/63/UE). All experimental protocols were approved by the internal ethics committee (Comitato Etico Sperimentazione Animale; CESA) and the Ministry of Health (Authorization n. 421/2017-PR issued on May 18, 2017).

### Western blotting

To examine the effect of FLX on MeCP2 levels, brain areas of *Mecp2* HET and WT mice were dissected out free-hand. Striatum, hippocampus and cortex were homogenized in 1% Sodium dodecyl sulphate (SDS) and total protein concentration was determined by the bicinchoninic acid protein assay (BCA Protein Assay Kit, Pierce Biotechnology, USA). Twenty µg protein/sample were loaded onto 10% acrylamide gels. The amount of protein was established by running increasing amount of protein on the gel (Supplementary Fig. 4). For the MW estimates of target proteins, Precision Plus Protein Dual Color Standards (BIO-RAD, Cat. #1610374) was loaded in one well of each gel. Proteins were separated by SDS polyacrylamide gel electrophoresis (SDS-PAGE) and electrophoretically transferred to a polyvinylidene difluoride (PVDF) membrane (Amersham, GE Healthcare).

Membranes were cut approximately in correspondence to the 50 kDa MW. Blots were blocked with 5% non-fat milk in tris-buffered saline (TBST) (20 mM Tris, pH 7.6, 140 mM NaCl, and 0.5% Tween-20) for 1 h at room temperature. Membranes containing proteins heavier than 50 kDa were incubated overnight at 4 °C with primary antibody against MeCP2 (MW = 75 kDa) diluted 1:2000 (Cell Signaling Technology, Cat. # 3456), which detects both isoforms 1 and 2 of the protein (datasheet). Membranes containing proteins lighter than 50 kDa were incubated with the antibody against glyceraldehyde 3-phosphate dehydrogenase (GAPDH; MW = 37 kDa) (Abcam, Cat. # Ab22555) diluted 1:20,000, as a loading control. After washing with TBST (5 × 5 min), anti-rabbit IgG, horseradish peroxidase (HRP)-linked antibody (Cell Signaling Technology, Cat. # 7074) were applied for 1 h at room temperature (1:5000 and 1:30,000 for MeCP2 and GAPDH, respectively). Primary antibodies were diluted in bovine serum albumin (BSA) 2% in TBST while the secondary antibody was diluted in 5% non-fat milk. The immune-positive protein bands were detected with a chemiluminescent home-made enhanced chemiluminescence (ECL) luminol/p-coumaric acid solution. Membranes were exposed to autoradiography films in the dark room (Hyperfilm ECL, Amersham GE Healthcare) then developed. Autoradiographs were scanned and signal intensity assessed with ImageJ (NIH) software. The ratio between MeCP2 and GAPDH for each sample was normalized to the WT mice average.

### Tissue fixation and sectioning

Twenty-four h after the last dose of FLX or vehicle, *Mecp2* HET and WT mice, were anaesthetized with 75 mg/kg ketamine plus 1 mg/kg medetomidine and perfused through the heart using a peristaltic pump (Mityflex, ANKO, Bradenton, FL) with Ice-cold 0.01 M phosphate buffer-saline (PBS Tablets; Sigma, Cat. # 5564) at 10 mL/min for 3 min followed by 4% para-formaldehyde (PAF) solution in PBS for 5 min. The brain was removed and post-fixed in 4% PAF for 4 h, at 4 °C. Brains were then dipped in a 20% sucrose solution and stored at 4 °C overnight. Brains were removed from the sucrose solution, damped with Kleenex, frozen in n-pentane at − 45 °C for 3 min and stored at − 80 °C until sectioning.

Brain sections (25 μm) for IHC were cut with a cryostat (CM1850-UV, Leica Microsystems, Buccinasco, Italy) at − 17 °C and mounted on gelatin-coated slides. Consecutive brain slices containing the areas of interest were mounted onto glass slides for 3,3′-diaminobenzidine tetrahydrochloride (DAB) staining.

### Immunohistochemistry

After drying-off overnight, slices were rehydrated with PBS and incubated with a solution containing 10 mM disodium citrate and 0.01% Tween-20, at 80 °C in a heater with agitation for 20 min. This step is necessary to unmask the MeCP2 epitopes to which the antibody binds. Once cooled to room temperature, sections were washed with 0.01% PBS and quenched from endogenous peroxidase with 0.3% H_2_O_2_ (Sigma, Milano, Italy). Sections were washed with 0.01 M PBS plus 0.5% Triton X-100 (PBT) and blocked with 5% NGS (Normal Goat Serum, Vector Laboratories, Segrate, Italy) and 2% BSA solution (Bovine Serum Albumin, Sigma, Milano, Italy) for 1 h. After 3 washes with PBT, sections were incubated for 48 h at 4 °C with monoclonal anti-rabbit MeCP2 (Cell Signaling Technology Cat# 3456S, RRID:AB 2143849) diluted 1:150 in blocking solution.

After incubation with the primary antibody, slices were rinsed in PBS and incubated with biotinylated horse anti-rabbit IgG for 1 h. Sections were then incubated with an avidin–biotin–peroxidase complex (Vectastain ABC Elite Kit) for 1 h, rinsed in PBS and reacted for about 12 min with the chromogen DAB and H_2_O_2_. After 3 washes in PBS, sections were coverslipped with DPX New Mounting Medium (Merck, Cat. # 100579). To avoid procedural bias, comparative brain slices of all genotype and treatment groups were processed simultaneously.

Staining was observed under a bright field microscope, the Virtual Slide Microscope (VS120) with a 2 × lens for the overview and 20 × lens for the acquisition (Olympus, Italy). MeCP2^+^ cells were counted in pre-defined areas of the dorsal (DSTR), ventral (VSTR) and lateral (LSTR) striatum, M1 and M2 motor cortices, prefrontal cortex (PFC) and CA3 sub-region of the dorsal hippocampus. Before the analysis, each image has been optimized by improving contrast/brightness and the background has been subtracted to better highlight the labeled protein Cell count biases were avoided by using automatic counting software (Image J) and custom-made macros to apply the same pre-defined functions: “Convert to Mask”, “Fill Holes”, “Watershed”, “Analyze Particles”, “size = 100 − Infinity and circularity = 0.00–1.00”. The automatic thresholding function of the software was used to distinguish the specific signal generated by MeCP2 from the background.

For each brain region, the MeCP2^+^ cells count was the mean of 2–5 adjacent slices/mouse. Specificity of the MeCP2 antibody is confirmed by the absence of specific staining and very low background signal in slices obtained from the brain of *Mecp2-null* mice (Supplementary Fig. 3).

A densitometric analysis of MeCP2^+^ cells in the same pre-defined area of the PFC, used to count the number of MeCP2^+^ cells, was done by the Image J software as described in detail elsewhere^57^. Briefly, images were converted to black and white and analysed by setting “Mean gray value” in the “Set measurement” window. Density was expressed as the average pixel intensity of the digitalized histochemical image.

### Drug treatment

Fluoxetine HCl (Casen, Recordati SL, Spain) was dissolved in pyrogen-free water (10 mL/kg). WT and *Mecp2* HET mice were injected intraperitoneally (i.p.) with 10 mg/kg FLX (as salt) once daily for 14 days and 24 h after the last dose of the chronic schedule they were anaesthetized and perfused intracardially with PAF solution as described above.

To assess whether chronic i.p. administration of FLX could have adverse effects on animal’s health, we monitored the weight gain during treatment, as this is a reliable indicator of their health. The *Mecp2* HET and WT mice, do not differ in weight and FLX did not affect the weight growth curve in either genotype^20^.

To assess the role of 5-HT in the ability of FLX to rescue motor deficits of *Mecp2* HET mice, mice were given 100 mg/kg pCPA ethyl ester hydrochloride as free base (Sigma-Aldrich, Milano, Italy) dissolved in pyrogen-free water. pCPA or water was given orally by gavage for three consecutive days simultaneously with FLX or vehicle i.p. (from day 15 to 17 from the first dose of FLX). The dose of pCPA was based on the results of previous studies showing full antagonism of the effect of SSRIs in the forced swimming test^53, 54^.

## Supplementary Information

Supplementary Information.
