# Supplementary material for: Fluoxetine increases brain MeCP2 immuno-positive cells in a female Mecp2 heterozygous mouse model of Rett syndrome through endogenous serotonin
Source: Sci Rep. 2021 Jul 19;11:14690. doi: 10.1038/s41598-021-94156-x (PMC8290043; doi:10.1038/s41598-021-94156-x)
Supplement: Supplementary file 1 — Supplementary Information. [file 41598_2021_94156_MOESM1_ESM.docx]

**Supplementary material**

**Fluoxetine increases brain MeCP2 immuno-positive cells in female *Mecp2* heterozygous mouse model of Rett syndrome through endogenous serotonin**

**Claudia Villani, Mirjana Carli, Anna Maria Castaldo, Giuseppina Sacchetti & Roberto William Invernizzi**

Laboratory Neurochemistry and Behavior, Neuroscience Dept., Istituto di Ricerche

Farmacologiche Mario Negri IRCCS, Via Mario Negri 2, 20156 Milano, Italy

Correspondence and requests for materials should be addressed to R.W.I. (email: [rinvernizzi@marionegri.it](mailto:rinvernizzi@marionegri.it); phone: +39 02 39014556)

**Supplementary Figure S1**

## Fig. S1 Effect of fluoxetine (FLX,10 mg/kg/day for 14 days) on the total number of cells in the prefrontal cortex (PFC), motor cortex M1 and M2, dorsal striatum (DSTR), ventral striatum (VSTR) and lateral striatum (LSTR) of WT and *Mecp2* HET (HET) female mice. The total number of cells (DAPI staining) was recorded in a subgroup of mice used to assess the effect of FLX on MeCP2 expression (Figs. 1 and 2 in the main text). DAPI (4′,6-Diamidine-2′-phenylindole dihydrochloride) immuno-positive cells (DAPI+) were measured in brain slices adjacent to those used for counting the number of MeCP2+ cells. DAPI is a dye that binds selectively to DNA forming strongly fluorescent DNA-DAPI complexes and is commonly used to establish the total number of cells in histological specimens. Histograms indicate the mean (± SEM) number of DAPI+ cells in the PFC, M1 and M2, CA3 and striatal subregions of WT and HET mice (6-8 mice/group). Black dots and squares represent individual data points. Blue and red columns indicate treatment with vehicle and FLX, respectively. There were no significant differences between groups.

**Supplementary Figure S2**

## Fig. S2 Effect of fluoxetine (FLX, 10 mg/kg/day for 14 days) on the number of MeCP2+ cells in the prefrontal cortex (PFC), motor cortex M1 and M2, dorsal striatum (DSTR), ventral striatum (VSTR) and lateral striatum (LSTR) of WT and *Mecp2* HET (HET) female mice. The number of MeCP2+ cells was normalized for the total number of cells (DAPI+) in slices adjacent to those used for counting MeCP2+ cells. Histograms indicate the mean (± SEM) percentages of MeCP2+ cells in the PFC, M1 and M2, CA3 and striatal subregions of WT and HET mice (6-8 mice/group). Black dots and squares represent individual data points. Blue and red columns indicate treatment with respectively vehicle (H_2_O) and FLX. ***P < 0.0001, **P <0.001 vs. WT-H_2_O; ^#^P < 0.05 vs. HET-H_2_O; (Sidak’s test; one-tailed).

**Supplementary Figure S3**

WT

KO

DAB staining staining


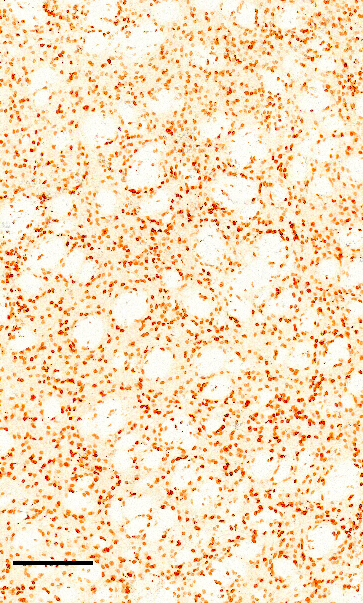

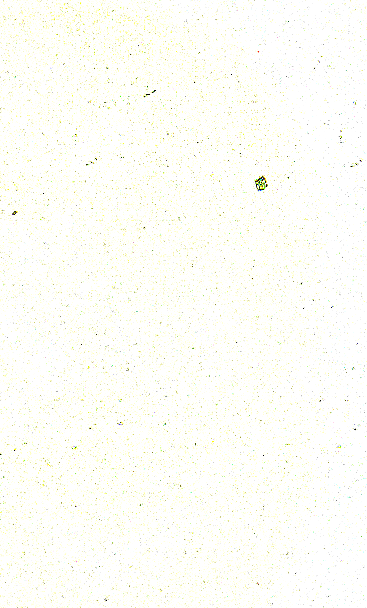


**Fig. S3** Specificity of the MeCP2 antibody was investigated by DAB immunohistochemistry in striatal slices of wild type (WT) and *Mecp2*-null (KO) mice aged 7-8 weeks. Brown dots are MeCP2+ cells. No specific staining was observed in *Mecp2*-null mice. Scale bar, 100 µM.

**Supplementary Figure 4**

**
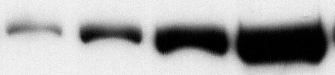
A**

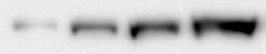
**B**

**Fig. S4** Linear relationship between the amount of proteins loaded onto the gel and chemiluminescence produced by MeCP2 specific immuno-positive bands. The corresponding blot is shown on top of panels A and B. Homogenates of the striatum (A) and cortex (B) containing 5-40 μg protein/sample were loaded onto the gel. The autoradiographs were scanned and signal intensity analyzed by Image J software (A).

As this method was subsequently replaced by ChemiDoc MP (Bio-Rad) scanning and ImageLab software for signal intensity, the cortical samples (B) were analysed by the new method. Graphs and correlation coefficients (Pearson r) were generated/calculated with the Prism software (version 7.02; [URL link](https://www.graphpad.com/scientific-software/prism/)).

**Supplementary Figure 5**

**A**


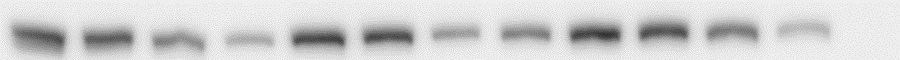

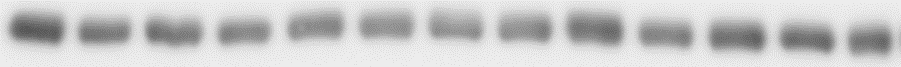

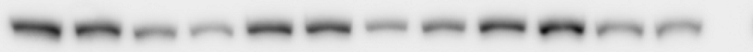

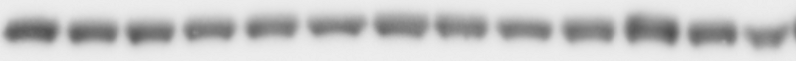


W/V W/F H/V H/F W/V W/F H/V H/F W/V W/F H/V H/F KO

GAPDH

**Blot 2**

MeCP2

MeCP2

GAPDH

**Blot 1**

**B** **____________________________________________________________**

Genotype Treatment MeCP2/GAPDH (mean ± SEM)

WT Vehicle 1.00 ± 0.07

WT Fluoxetine 1.10 ± 0.04

HET Vehicle 0.48 ±.0.04***

HET Fluoxetine 0.44 ± 0.07***

**Fig. S5** **Effect of fluoxetine on MeCP2 levels in the cortex of WT and *Mecp2* HET mice**

1. Representative immunoblots (full-length blots) of the MeCP2 and GAPDH proteins. Membranes were cut approximately in correspondence to the 50 kDa MW. The half membrane containing proteins heavier than 50 kDa was incubated with primary antibody against MeCP2, while the half membrane containing proteins lighter than 50 kDa was incubated with the antibody against GAPDH. W/V and W/F indicate WT mice receiving vehicle and fluoxetine. H/V and H/F indicate *Mecp2* HET (HET) mice receiving vehicle and fluoxetine. The specific MeCP2 immuno-positive band is absent in cortical samples of *Mecp2* null (KO) mice. see the main text for methodological details.
2. Normalized MeCP2 levels (Mean ± SEM of 6 mice/group) in wild type (WT) and *Mecp2* HET mice receiving fluoxetine or vehicle. ***p < 0.0001 vs. WT control group (Tukey’s test). Fgenotype (1,20) = 112.6, p < 0.0001; Ffluoxetine (1,20) =0. 289, p = 0.597; Finteraction (1,20) = 1.428, p = 0.246 (2-way ANOVA). The specific MeCP2 immuno-positive band (A) is absent in cortical samples of Mecp2-null (KO) mice

.

**Supplementary Figure 6**


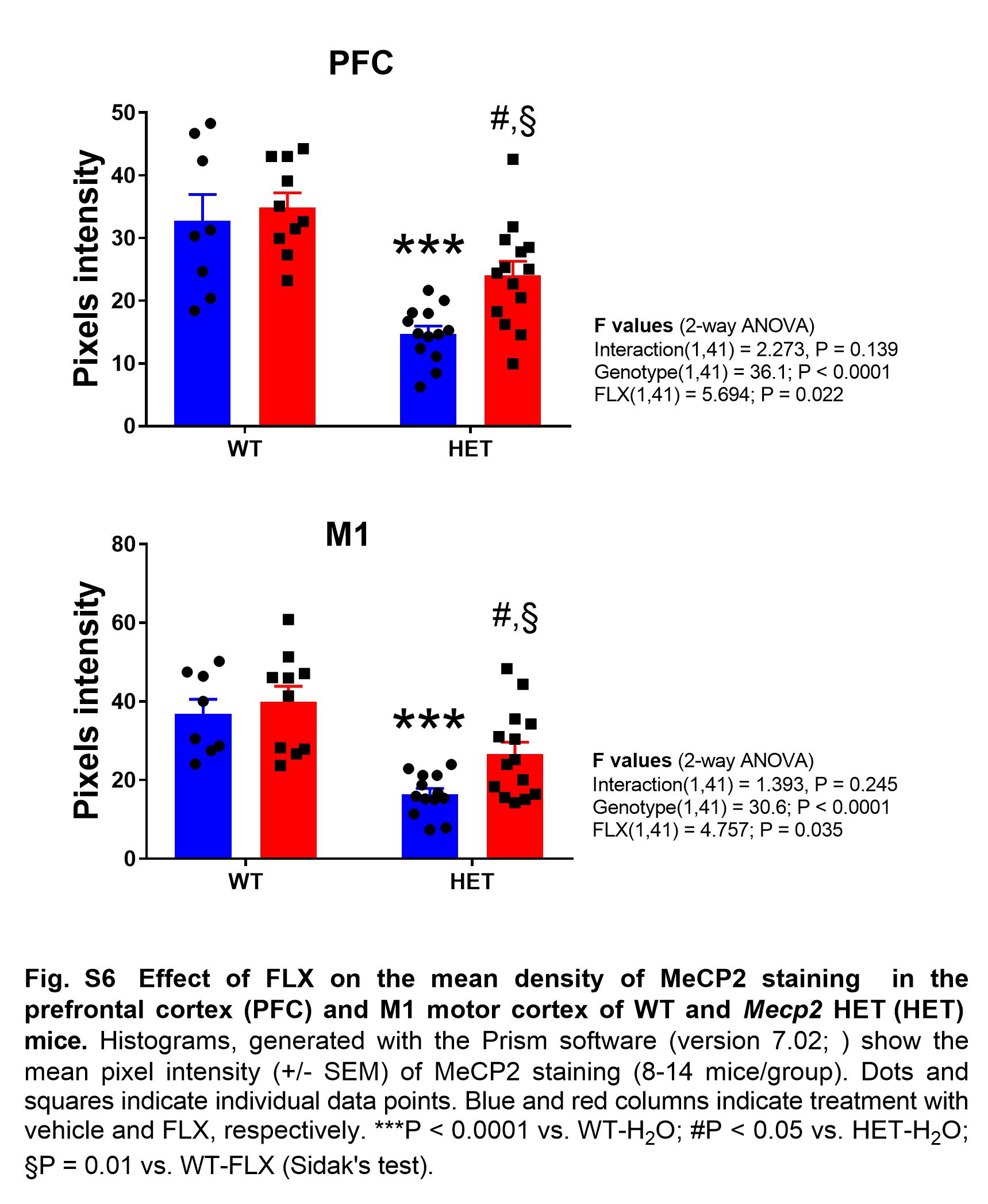


**Supplementary Table 1**

**Genotype Treatment** ***Mecp2*** (relative mRNA expression)

WT Vehicle 1.02 ± 0.08

WT Fluoxetine 1.24 ± 0.09

HET Vehicle 0.56 ±.0.04***

HET Fluoxetine 0.40 ± 0.04***

**Table S1** **Effect of FLX (10 mg/kg, once daily for 14 days) on Mecp2 gene expression in the striatum and cortex of *Mecp2* HET and WT mice** Striatal mRNA was isolated using Maxwell 16 LEV simplyRNA Tissue Kit (Promega), according to manufacturer’s instructions. First strand complementary DNA (cDNA) was synthesized from 1000 ng of total RNA using High-Capacity cDNA Reverse Transcription Kit (Applied Biosystem). RT-PCR was performed in triplicate for each sample on 7300 Real Time PCR System (Applied Biosystems). Reactions contained 100 ng of cDNA, 10 µL TaqMan Gene Expression Master Mix (Applied Biosystems), 1 µL specific Taqman probe and water to a final volume of 20 µL. PCR conditions were as follows: 50°C for 2 min, 95°C for 10 min, 40 cycles of 95°C for 15 s and 60°C for 60 s. Gene expression was normalized to *RpL19* as internal control. The following probes were used for the quantitative reverse transcription PCR: MeCP2 (Mm01193537_g1; Applied Biosystems); Rpl19 (Mm02601633_g1; Applied Biosystems). Data were analyzed using the 2^−(ΔΔCT)^ method expressed to WT-vehicle. Data are means ± SEM of 6 mice/group. FLX had no significant effect on *Mecp2* expression in WT and *Mecp2* HET mice. ***p < 0.0004 vs. WT (Tukey’s test). Fgenotype(1,20) = 98.98, p < 0.0001; Ftreatment(1,20) = 0.267, p = 0.610; Finteraction(1,20) = 8.51, p = 0.0085 (ANOVA)
